# A Study on Patient Safety Incidents and the Second Victim Phenomenon Among Healthcare Providers in Al-Ahsa, Saudi Arabia

**DOI:** 10.7759/cureus.49324

**Published:** 2023-11-24

**Authors:** Ola Mousa, Mohammed Sadeq Alghazal, Ali Abdullah AlBather, Amna Nasser Alhassan, Meriam Hussain Alamer, Zahra’a Taher Alghadeer, Salha Fayea Alasiri

**Affiliations:** 1 Department of Obstetrics and Gynecology Nursing, Faculty of Nursing, Minia University, Minya, EGY; 2 Department of Nursing, King Faisal General Hospital, Al-Ahsa, SAU; 3 Department of Service Excellence, Al-Ahsa Health Cluster, Al-Ahsa, SAU; 4 Department of Medical-Surgical Nursing, King Faisal General Hospital, Al-Ahsa, SAU; 5 College of Applied Medical Sciences, King Faisal University, Al-Ahsa, SAU; 6 Department of Health Informatics, King’s College London, London, GBR

**Keywords:** second victims, saudi arabia, patient safety, healthcare providers, adverse events

## Abstract

Background: A second victim (SV) is a healthcare worker who is traumatized by an unexpected adverse patient case, therapeutic mistake, or patient-associated injury that has not been anticipated. Often, the second victim experiences direct guilt for the harm caused to the patients. Healthcare organizations are often unaware of the emotional toll that adverse events can have on healthcare providers (HCPs) who can be harmed by the same incidents that harm their patients. Second victims (SVs) were present in 10.4% up to 43.3% of cases following an adverse event.

Aim: This study aims to examine the second victim phenomenon among healthcare providers at Al-Ahsa hospitals, its prevalence, symptoms, associated factors, and support strategies.

Methods: Four major public hospitals participated in this cross-sectional study. The study used the German standardized questionnaire "SeViD-I survey." The directors of the four hospitals sent invitations with links to participate to healthcare providers who had worked in their hospitals for over six months after completing their internship program.

Results: More than one-quarter of the respondents (90 (28%)) have been victims of a second victim incident before; of those, 63 (70%) have had it once, 12 (13.3%) twice, and 15 (16.7) repeatedly. In our study, the risk factors for a second victim only appeared in the male gender and were statistically significant. Strong reactivation of situations outside of the workplace was reported in 36 (40%) participants. Thirty-five (38.9%) participants reported reactivating the situation on the job site. Twenty-eight (31%) participants reported aggressive psychosomatic reactions (headaches and back pain). In 28 (31.1%) participants, sleep problems or excessive sleep needs were pronounced. The median of feeling symptoms was 7.2. As for supporting strategies, 64 (71.1%) respondents considered emotional support and crisis management to be very helpful. Sixty-six (73.3%) respondents found a safe chance to be very helpful.

Conclusion: The findings of this study indicate that healthcare providers in Al-Ahsa, Saudi Arabia, suffer from second victim traumatization at high rates. Several symptoms appear in the second victim, and most do not receive enough support.

## Introduction

It is important to provide safe, quality care in healthcare settings and to prevent injuries as much as possible. A healthcare-related adverse event affects eight out of every 100 patients in developing countries [1]. The expectation of perfection among healthcare workers is reinforced by societal opinion and expectations from others [2]. The term adverse events refers to hurts or complications caused by medical interferences that are unintentional and cannot be avoided [3]. Patients can suffer as a result of adverse events. The healthcare industry has approved standardized practices to examine incidents, and employees are becoming more alert of errors in the systems [4].

A second victim (SV) is a healthcare worker who is traumatized by an unexpected adverse patient case, therapeutic mistake, or patient-associated injury that has not been anticipated. Often, the second victim experiences direct guilt for the harm caused to the patients. A common feeling among clinicians is that they have been unsuccessful in treating their patients, considering their medical services and knowledge as "low" [5]. Traumatic patient care events in a healthcare setting can result in second victim symptoms. There are several types of events that can lead to mental stress for providers, for example, near misses, adverse events, and deaths. After the event, all second victims will have distinctive viewpoints, requirements, and reactions, and symptoms can occur at different times [2].

Healthcare organizations are often unaware of the emotional toll that adverse events can have on healthcare providers (HCPs) who can be harmed by the same incidents that harm their patients [6]. Medical errors are human; we need to break the silence surrounding them and their consequences not by blaming healthcare providers without considering their well-being [6].

As a result of stressful patient-related events, hospital workers face many challenges [7]. As a result of adverse events, patients are the first victims, followed by their families [8]. The second victim will be the healthcare professionals, and the third victim will be the hospital's reputation [5]. In using the expression victim, double concerns arise: implying inactiveness or stigmatizing involved healthcare providers [9,10]. Responsibility is not denied, but attention is needed [11].

Second victims (SVs) were present in 10.4% up to 43.3% of cases following an adverse event [12]. At least half of healthcare providers suffer from the effect of being an SV [13]. In fact, there is always an SV when a serious adverse event occurs [14]. Furthermore, misuse claims in hospitals are related to "surgical" or "infusion errors," while the majority of claims in outpatient care relate to "unnoticed" or "late diagnosis" [15]. In Saudi Arabia, no clear information was available about the second victim phenomenon.

The expectancy of excellence in medicine leads to medical errors being viewed as personal failures by healthcare providers [5]. The second victim syndrome affects almost all providers at some point in their careers. The symptoms are more like a variety of feelings and symptoms than a binary presence or absence. It is possible to experience these symptoms even if they do not meet the full definition of a syndrome [16].

There is often a sense of guilt among second victims, and they may disbelieve in their own abilities and knowledge as the consequences of an adverse event [17]. They may also experience psychological and psychosomatic symptoms and consider changing careers [18]. As a result, they take more sick leave [19]. In addition, the second victim tends to move to a different department [20]. It is even possible for them to leave their profession [21].

There is an increased risk of depression and suicide associated with severe or long-lasting symptoms of the second victim phenomenon. Those who have faced adverse outcomes in the prior two years have a 1.64 times greater likelihood of contemplating suicide the following year [22]. According to Shanafelt et al. (2011), those who consider suicide are 3.4 times more likely to have reported a person-perceived medical error in the past three months [23].

Aim

The aim of this study is to investigate the prevalence, symptoms, associated factors, and support strategies of second victimization among healthcare providers in Al-Ahsa hospitals.

Objectives

The objectives of this study are to verify the prevalence of the second victim phenomenon, determine the physical and psychological symptoms suffered by healthcare providers after adverse events throughout patient care, detect the factors accompanying the second victim phenomenon in Al-Ahsa public hospitals, and recognize the experiences of the quality of support taken and the desired forms of support after adverse events.

## Materials and methods

Area and design of the study

Four major public hospitals participated in this cross-sectional study: King Faisal General Hospital, Bin Jalawi Hospital, King Fahad Hofuf Hospital, and Maternity and Children's Hospital (MCH) Hospital, Saudi Arabia. Researchers included all registered healthcare providers (HCPs) with the Saudi Commission for Health Specialties in Al-Ahsa (N=3,372) as given by the health cluster.

All registered healthcare providers in the Saudi Commission for Health Specialties who have completed at least more than six months of work experience in hospitals were eligible to participate, regardless of their race, gender, age, or educational background.

Principal officers in the chosen hospitals were provided consent forms to distribute to healthcare providers via email once necessary approvals were obtained from each hospital.

In this study, all healthcare providers were invited to participate. A total of 321 participants completed the survey.

For ethical consideration, the Institutional Review Board of King Fahad Hospital, Hofuf, approved this study (IRB log number: 14-EP-2023).

Study instrument

The study used the German standardized questionnaire "SeViD-I survey" [24]. The form contains three parts and 40 questions. The first part was to address the overall being involved in a second victim case (seven questions). In regard to the symptoms part, participants responded with a 3-point ordinal scale (strongly pronounced, weakly pronounced, and not pronounced) for the support strategies and a 4-point ordinal scale (very helpful, rather helpful, rather not helpful, and not helpful) for the support strategies (13 questions).

Prior to the main study, healthcare providers were notified of the study's goal and the anonymity of all information they submitted. In addition, they were made aware that involvement in this study is optional and that the results will strictly be used for academic research only. The respondents were then required to fill out self-administered questionnaires.

Design and conduct of the SeViD-I survey

For the purpose of conducting the survey, the commercial application Google Forms (Google, Inc., Mountain View, CA) was used. The directors of the four hospitals sent invitations with links to participate to healthcare providers who had worked in their hospitals for over six months after completing their internship program. The period of the study spanned from the 20th of March to the 20th of May 2023. After two, four, and six weeks, a reminder was sent, and it took eight weeks for the survey to close. Data collection was anonymized completely. Both the invitation letter and the reminder contained a summary of the study's goals, a brief description of the second victim phenomenon, and a link to the online form. Data regarding sociodemographic characteristics, formal medical education, and years of work experience were reported.

A pilot study was conducted to study questionnaire validity and reliability. Cronbach's alpha reliability scores for the survey dimensions ranged from 0.73 to 0.89.

Only those participants who indicated that they had experienced the specific incident were shown items from the symptom domain. Answering each question was mandatory. Based on their responses to the 20 items in this domain, a sum score was calculated to estimate participants' symptom load. Assigning 1 to "strongly pronounced" and 0.5 to "weakly pronounced" (input zero for "don't know" and "not at all"). Afterward, participants' sum scores were calculated. This new variable was dichotomized based on the median (7.2) to establish groups for low and high symptom loads. A low symptom load was considered when it was 7.1 or less, and a high symptom load was considered when it exceeded 7.2.

## Results

Table 1 shows the sociodemographic data of the study participants. Three hundred twenty-one participants fully completed the survey. In terms of age, nearly one-third of the participants were between 31 and 35 years. There were 269 (83.8%) females in the study. The majority of the respondents (277 (86.3%)) were Saudi nationals. More than two-thirds (244 (76%)) were nurses. One-third of the participants (106 (33%)) had experience as healthcare providers for more than 10 years.

**Table 1:** Sociodemographic data (N=321)

Item	Number	Percentage (%)
Age		
20-25 years	37	11.5
26-30 years	85	26.5
31-35 years	106	33
36 years and above	93	29
Gender		
Female	269	83.8
Male	52	16.2
Nationality		
Non-Saudi	44	13.7
Saudi	277	86.3
Profession		
Nurse	244	76
Other	42	13.1
Physician	35	10.9
Work experience in total as a healthcare provider		
0-1 year	47	14.6
2-5 years	89	27.7
5-10 years	79	24.6
More than 10 years	106	33

For perceptions and experiences of a second victim incident, Table 2 shows that more than three-quarters (248 (77.3%)) of the respondents were unaware of the term. The majority of respondents were unsure of the exact meaning of the term "second victim phenomenon." Nearly three-quarters (231 (72%)) had no experience of second victim syndrome. There were reports of a death caused by an event (55 (17.1%)), while an event that resulted in the loss of an organ or function of an organ was reported by nine (2.8%) respondents and an event that resulted in temporarily severe harm was reported by 24 (7.5%).

**Table 2:** Perceptions and type of participation

Item	Number		Percentage (%)
Is the term "second victim" familiar to you?			
Yes, I knew it		73	22.7
No		248	77.3
Is the second victim phenomenon defined as the psychological and social harm affecting healthcare providers involved in patient/colleague incidents?			
No		40	12.5
Yes		128	39.9
I don't know exactly		153	47.6
Have you been involved in any of the following events?			
An event that resulted in a death		55	17.1
An event that resulted in a loss of an organ or function of an organ		9	2.8
An event that resulted in temporarily severe harm		24	7.5
An event that resulted in no harm		2	0.6
None of the above/not applicable		231	72
Affected person/facility			
Colleague		20	6.2
Facility/property		10	3.1
Not applicable		200	62.3
Patient		84	26.2
Visitor		7	2.2
Level of your involvement			
Direct (your act caused the error)		9	2.8
Indirect (your act had a minimum influence on the error)		23	7.1
Not applicable		231	72
Semidirect (your act contributed to the error)		10	3.1
Witness only		48	15

**Figure 1:**
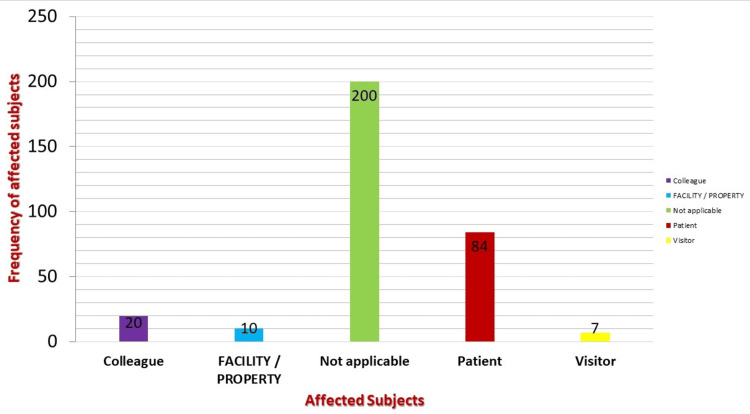
Affected person or facility

The affected person or facility is shown in Figure 1. There is no doubt that patients (26% (84/321)) followed by colleagues (6.2% (20/321)) were reported as the most frequently affected persons.

**Figure 2:**
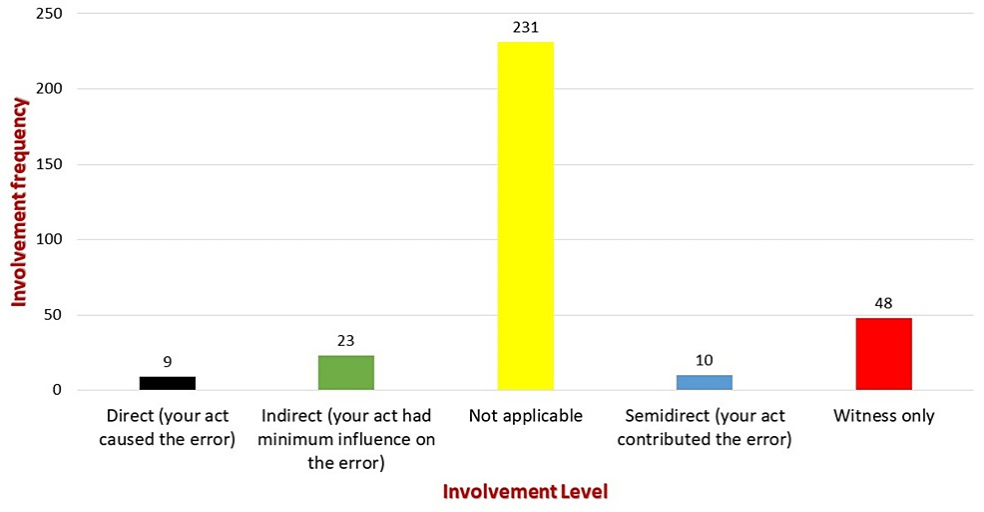
Participants' level of involvement

Figure 2 illustrates the participants' level of involvement. Forty-eight (15%) participants were witnesses only, 23 (7.2%) had an indirect level of involvement, 10 (3.1%) had semidirect involvement in the event, and nine (2.8%) had direct involvement in the event.

Table 3 illustrates the general experience with the second victim phenomenon. Regarding the time to full recovery after key incidents as perceived by the individual, more than one-quarter (27 (30%)) of the participants were not aware of when they had fully recovered. Nearly one-quarter (22 (24.4%)) had no symptoms because they were witnesses only, although some witnesses had symptoms. Nearly one-quarter (22 (24.4%)) had symptoms for more than one month, while 11 (12.2%) had symptoms for less than one month. As for the prevalence of the second victim phenomenon during the last year of work, 63 (70%) participants reported experiencing it once, while 12 (13.3%) reported experiencing it twice. Death accounted for 34 (37.8%) key incidents, followed by medication prescription and administration problems with 27 (30%) and falling with 18 (20%). In terms of seeking help and support, 50% did not do so. As regards the types of groups supporting after the key incident, nearly half (43 (48.8%)) did not receive any assistance, while 17 (18.9%) received assistance from authorized staff or department heads.

**Table 3:** Experiencing the second victim phenomenon IUFD: intrauterine fetal demise

Item	Number		Percentage (%)
Time to full recovery after key incidents as perceived by the individual (n=90)			
I haven't recovered yet		8	8.9
I'm a witness ("no symptoms")		22	24.4
Less than one month		11	12.2
More than one month		22	24.4
I don't know exactly		27	30
12-month prevalence of second victim experience			
1 incident		63	70
2 incidents		12	13.3
More than 2		15	16.7
Type of key incident			
Complications of surgery		2	2.2
Death		34	37.8
Fall		18	20
Injury		7	7.8
Intubation		1	1.1
IUFD for pregnant women		1	1.1
Medication administration problem		27	30
Seek for support after key incident			
No		45	50
Yes		45	50
Supporting groups after a key incident			
Colleagues		16	17.8
Didn't receive		43	48.8
Friends		10	11.1
Head of the department or authorized person		17	18.9
Psychiatrist		4	4.4

**Figure 3:**
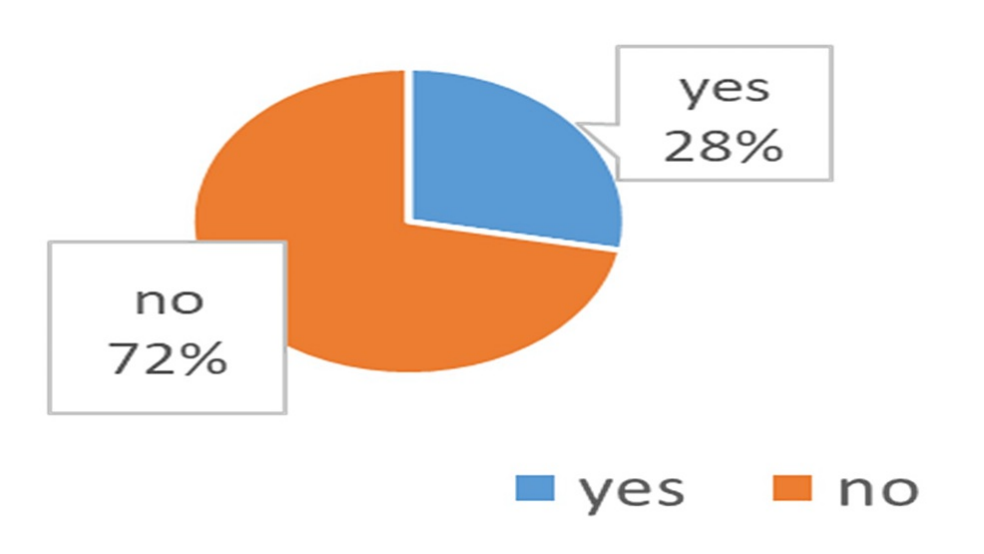
Overall prevalence of the second victim phenomenon

**Figure 4:**
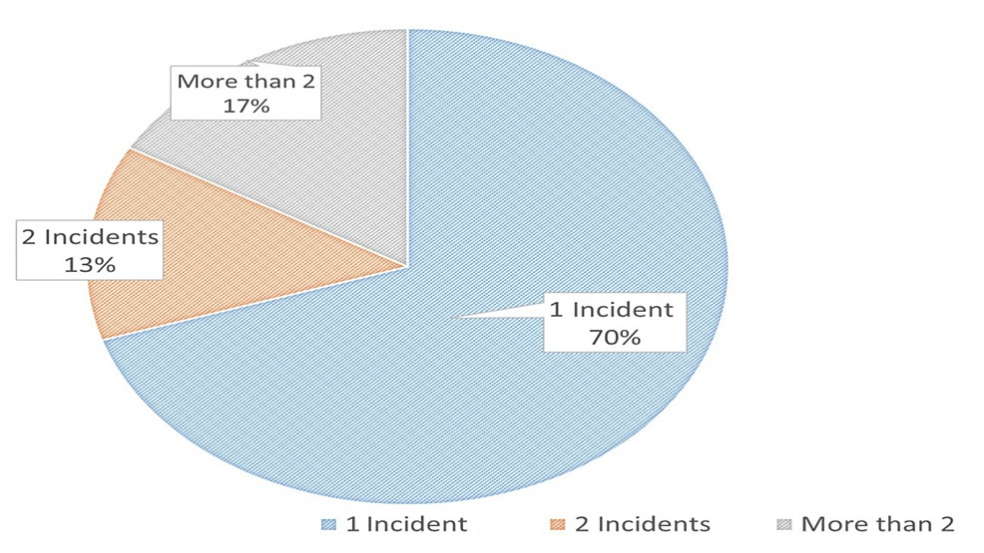
Prevalence of the second victim phenomenon during the year

Figure 3 and Figure 4 illustrate the prevalence of the second victim phenomenon among the study participants and the number of experiences of the phenomenon throughout the year. More than one-quarter of the respondents (90 (28%)) have been victims of a second victim through the year; of these, 63 (70%) have had it once, 12 (13%) twice, and 15 (17%) repeatedly.

Table 4 illustrates the symptoms of the second victim phenomenon. Fear of social exclusion from colleagues was strongly pronounced in 12 (13.3%) of the participants, worry of loss of job in 20 (22.2%), lethargy in 24 (26.7%), and depressed mood in 30 (33.3%). Concentration problems strongly appear in 28 (31.1%) participants. Reactivation of situations outside the job site was strongly pronounced in 36 (40%). Reactivation of the situation at the job site was strongly pronounced in 35 (38.9%). Aggressive, risky behavior was strongly pronounced in six (6.7%). Defensive, overprotective behavior was strongly pronounced in 25 (27.8%). Psychosomatic reactions (headaches and back pain) appeared with 28 (31.1%). Sleep problems or excessive sleep needs were pronounced in 28 (31.1%) participants. The median of feeling symptoms was 7.2.

**Table 4:** Symptoms of the second victim phenomenon

Item	Number		Percentage (%)
Fear of social exclusion from colleagues			
Not pronounced		59	65.6
Strongly pronounced		12	13.3
Weakly pronounced		19	21.1
Worry of loss of the job			
Not pronounced		48	53.3
Strongly pronounced		20	22.2
Weakly pronounced		22	24.4
Lethargy			
Not pronounced		44	48.9
Strongly pronounced		24	26.7
Weakly pronounced		22	24.4
Depressed mood			
Not pronounced		29	32.2
Strongly pronounced		30	33.3
Weakly pronounced		31	34.4
Concentration problems			
Not pronounced		41	45.6
Strongly pronounced		28	31.1
Weakly pronounced		21	23.3
Reactivation of situation outside the job site			
Not pronounced		32	35.6
Strongly pronounced		36	40
Weakly pronounced		22	24.4
Reactivation of the situation at the job site			
Not pronounced		29	32.2
Strongly pronounced		35	38.9
Weakly pronounced		26	28.9
Aggressive, risky behavior			
Not pronounced		71	78.9
Strongly pronounced		6	6.7
Weakly pronounced		13	14.4
Defensive, overprotective behavior			
Not pronounced		40	44.4
Strongly pronounced		25	27.8
Weakly pronounced		25	27.8
Psychosomatic reactions (headaches and back pain)			
Not pronounced		45	50
Strongly pronounced		28	31.1
Weakly pronounced		17	18.9
Difficulties to sleep or excessive need to sleep			
Not pronounced		36	40
Strongly pronounced		28	31.1
Weakly pronounced		26	28.9
This event led to the use of substances (alcohol/drugs)			
Not pronounced		85	94.4
Strongly pronounced		2	2.2
Weakly pronounced		3	3.3
Sense of shame			
Not pronounced		59	65.6
Strongly pronounced		14	15.6
Weakly pronounced		17	18.9
Feelings of guilt			
Not pronounced		37	41.1
Strongly pronounced		29	32.2
Weakly pronounced		24	26.7
Lower self-confidence			
Not pronounced		53	58.9
Strongly pronounced		18	20
Weakly pronounced		19	21.1
Social isolation			
Not pronounced		54	60
Strongly pronounced		19	21.1
Weakly pronounced		17	18.9
Anger against others			
Not pronounced		53	58.9
Strongly pronounced		16	17.8
Weakly pronounced		21	23.3
Anger against oneself			
Not pronounced		44	48.9
Strongly pronounced		21	23.3
Weakly pronounced		25	27.8
Desire to get support from others			
Not pronounced		49	54.4
Strongly pronounced		22	24.4
Weakly pronounced		19	21.1
Having a desire to better understand the incident			
Not pronounced		36	40
Strongly pronounced		38	42.2
Weakly pronounced		16	17.8
Mean		7.5	
Median		7.2	

The supporting strategy used by the victim is shown in Table 5. Approximately 54 (60%) participants believed that immediate recovery time was very helpful. Counseling services, including psychological and psychiatric services, were considered very helpful by 47 (52.2%) respondents. Discussion of emotional and ethical issues (51.1%) was found to be very helpful. The majority of respondents (56 (62.2%)) found detailed explanations about procedures (e.g., cause-and-effect analysis and notification of incidents) to be very helpful. According to 59 (65.6%) respondents, peer-to-peer support is very helpful. Emotional support provided informally and immediate crisis management were considered very helpful by 64 (71.1%) of them. In terms of strategy, safe chance earned the highest score for providing explanations in order to prevent making the same mistake again, which was considered very helpful by 66 (73.3%).

**Table 5:** Support strategies

Item	Number		Percentage (%)
Immediate time-out to recover			
Not helpful		4	4.4
Rather helpful		30	33.3
Rather not helpful		2	2.2
Very helpful		54	60
Access to counseling, including psychological/psychiatric services			
Not helpful		7	7.8
Rather helpful		36	40
Rather not helpful		0	0
Very helpful		47	52.2
Possibility of discussing issues of emotion and ethics			
Not helpful		10	11.1
Rather helpful		28	31.1
Rather not helpful		6	6.7
Very helpful		46	51.1
In-depth knowledge about procedures (e.g., root cause analysis and report of incidents)			
Not helpful		5	5.6
Rather helpful		28	31.1
Rather not helpful		1	1.1
Very helpful		56	62.2
Formal peer-to-peer support			
Not helpful		5	5.6
Rather helpful		25	27.8
Rather not helpful		1	1.1
Very helpful		59	65.6
Informal emotional support			
Not helpful		5	5.6
Rather helpful		21	23.3
Rather not helpful		0	0
Very helpful		64	71.1
Prompt debriefing/crisis intervention			
Not helpful		4	4.4
Rather helpful		20	22.2
Rather not helpful		2	2.2
Very helpful		64	71.1
Supportive guidance for continuing clinical duties			
Not helpful		4	4.4
Rather helpful		20	22.2
Rather not helpful		5	5.6
Very helpful		61	67.8
Help to communicate with patients			
Not helpful		8	8.9
Rather helpful		18	20
Rather not helpful		9	10
Very helpful		55	61.1
A clear description of what roles are expected after an incident has occurred			
Not helpful		4	4.4
Rather helpful		24	26.7
Rather not helpful		3	3.3
Very helpful		59	65.6
Participate actively in the resolution of this incident			
Not helpful		5	5.6
Rather helpful		26	28.9
Rather not helpful		6	6.7
Very helpful		53	58.9
Contributing insights to prevent similar events in the future is a safe opportunity			
Not helpful		4	4.4
Rather helpful		17	18.9
Rather not helpful		3	3.3
Very helpful		66	73.3
Obtaining legal advice after an incident is possible			
Not helpful		6	6.7
Rather helpful		23	25.6
Rather not helpful		5	5.6
Very helpful		56	62.2

A second victim's risk factors are shown in Table 6. An analysis of the impact of gender, age, years of work, specialization, and nationality on the second victims' symptom load was conducted using a binary logistic regression model. Among the variables in this model, only the male gender had a statistically significant association with a high symptom load, with an odds ratio of 1.4 (p=0.03).

**Table 6:** Factors contributing to second victimization *Correlation is significant at the 0.05 level (two-tailed).

Factors affecting the second victim phenomenon		
Age	Pearson correlation	0.060
	Significance (two-tailed)	0.286
Gender*	Pearson correlation	0.121^*^
	Significance (two-tailed)	0.030
Work experience in total as a healthcare provider	Pearson correlation	0.049
	Significance (two-tailed)	0.378
Nationality	Pearson correlation	-0.193
	Significance (two-tailed)	0.069

## Discussion

It is important to understand the second victim phenomenon and its associated factors and suitable support strategies for victims. Within this context, this study is one of the limited number of studies carried out to investigate the prevalence, risk factors, and predisposition of second victimization among healthcare providers in Al-Ahsa hospitals in Saudi Arabia.

When the results obtained from the study were examined, the prevalence was more than one-quarter of the healthcare providers, which is considered a huge percentage and needs more attention. It has been found that second victim trauma is common among healthcare professionals, particularly in the USA, and the impact on patients may be high, as well as on healthcare specialists, coworkers, and healthcare organizations [12]. Saudi Arabia has limited data, implying the need for more research and campaigns there.

It was determined in the study that more than three-quarters (77.3%) of the respondents were unaware of the term. According to the German survey, nine out of 10 participants had never heard the term "second victim." This does not necessarily indicate that these healthcare providers did not experience trauma at work, however; it is evident that the trend exists. Comparatively, the study by Edrees et al. in 2011 reported that 46% (a total of 139 Johns Hopkins male nurses participated in the study) knew about the term and its definition [14]. There is one possible reason for this: healthcare professionals' trauma is not mentioned in Saudi medical schools and hospitals, which do not offer specialty training or support programs. Also, it is possible to explain the differences in results by the fact that the kingdom and some hospitals, in particular, have begun to develop policies concerned with the quality of healthcare, including attention to the health team itself.

Our study results revealed that patients (26%) followed by colleagues (6.2%) were reported as the most frequently affected persons. Similar results were reported by Strametz et al. in the Federal Republic of Germany, in which patient harm incidents (34%) and unexpected deaths and suicides of patients (35%) were listed as prominent events [24]. The most traumatizing incidents in the present study involved direct patient harm. A study from 2007 by Waterman et al. concluded that one-third of physicians participating in near misses suffered from usual second victim trauma [13]. This correspondence in results is expected because healthcare providers deal with patients in the first place, and therefore, direct error with patients is expected.

The frequency of single or repeated second victim traumatic experiences realized in our findings among healthcare providers was 28% last year. The result of the study carried out by Strametz et al. found the prevalence of second victim experiences to be high (overall: 59%) among German physicians in internal medicine, with 35% of the physicians experiencing them in the last 12 months [24]. The study of Scott et al. in 2010 among different health specialists, undergraduates included, found an annual occurrence of 30% [25], the study by Lander et al. in 2006 among surgeons showed a six-month prevalence of 10% [26], and the study by Wolf et al. conducted in 2000 found an annual prevalence of 43.1% among various healthcare professionals [27]. It is possible that these different results can be explained by the fact that most of these studies included different nationalities and specialties. There is no clear indication that prevalence differs significantly with any factor except gender, according to the results.

In the present study, the duration of recovery according to participants' perceptions varied. Nearly one-third (36.7%) reported up to one month, and 63.4% stated more than one month. A study by Gazoni et al. reported that 19% of traumatized physicians do not fully recover [18]. Another study by Burlison et al. reported that the second victim phenomenon could lead to leaving of the profession as a result [28].

In contrast, the self-estimated time to recover fully after the key event was described as up to one month by 72% and more than one month by 28% of the participants [24]. To explain these differences in the results, we need more research with a focus on the characteristics of healthcare providers or individual factors in dealing with errors and the time needed for full recovery after it.

In the present study, approximately 60% of the participants believed that immediate recovery time was very helpful. Informal emotional support and prompt debriefing/crisis intervention were considered very helpful by 71.1% of them. In terms of strategy, safe chance received the highest score for contributing insights to avoid similar actions in the future, which was considered very helpful by 73.3%.

Our study shows that most traumatized healthcare providers who received support obtained it from the head of the department or authorized person (18.9%), followed by colleagues (17.8%). Unfortunately, 48.8% did not receive any support. Along the same line, the study conducted by Scott et al. reported that up to 40% might not receive the right support they need [25].

A high symptom load was only associated with male sex in this study. By contrast, Strametz et al. found a risk factor associated with female gender [24]. There are gender-related differences in the second victim phenomenon described in published studies. According to the quantitative review by Tolin and Foa from 2006, females are more likely than males to meet post-traumatic stress disorder criteria [29]. In spite of this, they are less likely to experience potentially traumatic events. The findings of Kaldjian et al., Muller and Ornstein, and Wu et al. indicate that females are more sensitive to stress after traumatizing events (e.g., feeling of guilt and worrying about losing their confidence and reputation), but they handle the situation more constructively than men (e.g., more inclined to discuss errors or support changes in practice) [24,29-32]. The studies' findings may be influenced by the nature of working as a foreign healthcare provider or by the responsibilities that males feel at work. To explain differences, further research should focus on individual factors such as aspects of personality, elements of traumatizing circumstances, or influences of the environment.

Limitations

There may be a variety of limitations to our findings. The survey had a low response rate and a high dropout rate that increased with the duration. Potential participants may have been afraid to reveal that events failed to go as planned, and as a result, response rates may have been negatively affected. The workplace still has a recrimination and blame culture. The unidentified nature of the data gathered also prevents us from excluding multiple participation from certain participants.

## Conclusions

This study reveals the high prevalence of second victim traumatization among healthcare providers in Al-Ahsa, Saudi Arabia. There are many symptoms that appear in second victims, and there is a lack of support for most of the participants. The study revealed the importance and the effect of the phenomenon on healthcare teams. However, there is a gap in the knowledge about the cause and the effect on the quality of care that the affected person anticipated. Further research is needed in this field.
